# Cross-species identification of a plasma microRNA signature for detection, therapeutic monitoring, and prognosis in osteosarcoma

**DOI:** 10.1002/cam4.438

**Published:** 2015-03-17

**Authors:** Wendy Allen-Rhoades, Lyazat Kurenbekova, Laura Satterfield, Neha Parikh, Daniel Fuja, Ryan L Shuck, Nino Rainusso, Matteo Trucco, Donald A Barkauskas, Eunji Jo, Charlotte Ahern, Susan Hilsenbeck, Lawrence A Donehower, Jason T Yustein

**Affiliations:** 1 Department of Pediatrics, Baylor College of MedicineHouston, Texas; 2 Department of Virology and Microbiology, Baylor College of MedicineHouston, Texas; 3 Department of Preventive Medicine, Keck School of Medicine of the University of Southern CaliforniaLos Angeles, California; 4 Biostatistics and Informatics Shared Resource, The Dan L. Duncan Cancer Center, Baylor College of MedicineHouston, Texas

**Keywords:** Biomarker, microRNA, mouse model, osteosarcoma, plasma

## Abstract

Osteosarcoma (OS) is the primary bone tumor in children and young adults. Currently, there are no reliable, noninvasive biologic markers to detect the presence or progression of disease, assess therapy response, or provide upfront prognostic insights. MicroRNAs (miRNAs) are evolutionarily conserved, stable, small noncoding RNA molecules that are key posttranscriptional regulators and are ideal candidates for circulating biomarker development due to their stability in plasma, ease of isolation, and the unique expressions associated with specific disease states. Using a qPCR-based platform that analyzes more than 750 miRNAs, we analyzed control and diseased-associated plasma from a genetically engineered mouse model of OS to identify a profile of four plasma miRNAs. Subsequent analysis of 40 human patient samples corroborated these results. We also identified disease-specific endogenous reference plasma miRNAs for mouse and human studies. Specifically, we observed plasma miR-205-5p was decreased 2.68-fold in mice with OS compared to control mice, whereas, miR-214, and miR-335-5p were increased 2.37- and 2.69-fold, respectively. In human samples, the same profile was seen with miR-205-5p decreased 1.75-fold in patients with OS, whereas miR-574-3p, miR-214, and miR-335-5p were increased 3.16-, 8.31- and 2.52-fold, respectively, compared to healthy controls. Furthermore, low plasma levels of miR-214 in metastatic patients at time of diagnosis conveyed a significantly better overall survival. This is the first study to identify plasma miRNAs that could be used to prospectively identify disease, potentially monitor therapeutic efficacy and have prognostic implications for OS patients.

## Introduction

Osteosarcoma (OS) is the most common primary bone tumor and is seen predominantly in children and young adults 1. The 5-year overall survival is approximately 65% and the best predictor of long-term survival is the absence of metastatic disease at diagnosis 1. Unfortunately, even those patients who achieve remission after aggressive multimodal therapy have a high risk of recurrence and the disease becomes increasingly difficult to cure after recurrence. All patients require long-term monitoring, which consists of radiographic studies that can be ambiguous to interpret after therapy. Presently, there is no reliable, noninvasive, specific, and efficient biomarker to monitor tumor response and provide surveillance for tumor recurrence after the completion of therapy.

MicroRNAs (miRNAs) are stable, small noncoding RNA molecules that are conserved across multiple species and are posttranscriptional regulators of protein expression implicated in regulating physiologic and pathologic processes, including cancer 2,3. They are ideal candidates for plasma biomarker development due to their stability in plasma, ease of isolation and detection, and the unique expression patterns associated with disease states 4–6.

One reason for the difficulty in developing a biomarker for OS is the rarity of the disease as well as the paucity of adequately annotated clinical samples needed for appropriate determination of disease and stage-specific biomarkers. One effective method to overcome this problem is to utilize animal models and translate those findings to humans, which can only be performed when there is cross-species conservation of the biomarker in question 7. In this project, we used a novel genetically engineered mouse model (GEMM) of OS to discover a signature plasma miRNA profile that can identify the presence of OS. Besides the ability to detect and identify diseased state, the profile demonstrates the potential to monitor tumor response to chemotherapy in an orthotopic mouse model utilizing this miRNA profile. This plasma miRNA profile was further translated to human patient samples. Finally, we provide evidence that plasma miR-214, a member of the signature, has prognostic significance in human OS patient plasma samples. This is the first report of translating miRNA discoveries from a mouse model into human samples in OS, and shows direct applications toward assessing tumor detection and implications for clinical prognosis in high-risk OS patients.

## Materials and Methods

### Mouse model

A GEMM of OS used in this project was developed in our laboratory 8. It contains a germline 2.3 kb Col-*α*1(I) promoter region upstream of the Cre-recombinase gene along with one or both *Trp53* alleles flanked by LoxP recombination sites 9. In this model osteoblast-specific deletion of *Trp53* results primarily in OS. Mice were maintained in barrier facilities at Baylor College of Medicine (BCM) and provided with food and water ad libitum. All animal experiments were conducted according to institutional animal care and use committee (IACUC) protocols after approval was obtained from the BCM Institutional Review Board (BCM Animal Protocols AN-336 and AN-5225).

### Mouse tissue and plasma samples

Genotypic analysis was performed by PCR-based screening for Cre and wild-type *p53* to identify wild-type mice (p53+/+;Cre+/−), heterozygous mice (p53F/+;Cre+), and homozygous mice (p53F/F;Cre+) as described previously 10. Once mice developed a tumor, they were sedated with isoflurane per IACUC protocols and blood was obtained by cardiac puncture and placed into ethylene-diamine-tetra-acetic acid (EDTA) blood collection vials. Wild-type littermates were randomly selected and sacrificed for control samples. The blood samples were centrifuged at 1300*g* for 25 min at 4°C. The resultant plasma was isolated and centrifuged at 1000*g* for 5 min to remove debris. All plasma was stored at −80°C until further processing. Necropsy was completed and slides were prepared with hematoxylin and eosin stains to confirm an OS diagnosis.

### Extraction of circulating RNA from mouse plasma samples

Plasma samples were thawed and 15 *μ*L plasma was passed through a 0.22 *μ*m filter to remove any leftover cellular debris. RNA extraction was performed on 10 *μ*L of plasma. Total RNA, including miRNA, was extracted using guanidine thiocyanate followed by a solid phase extraction on a silica spin column. Briefly, 250 *μ*L of Qiazol lysis reagent (Qiagen, Germantown, MD) was added to 10 *μ*L of plasma, vortexed and incubated for 5 min at room temperature. To reduce the loss of small RNA molecules, 0.625 ng of a carrier RNA from the bacteriophage MS2 was added to the denatured samples (Roche Applied Science, Basel, Switzerland). Subsequently, 50 *μ*L of chloroform was added to the denatured samples, vortexed, incubated at room temperature for 2 min, and then centrifuged at 12,000*g* for 15 min at 4°C. The aqueous phase was removed and mixed with 1.5× volume of ethanol and miRNAs were isolated from the aqueous phase using the miRNeasy silica spin columns (Qiagen) per manufacturer instructions with the addition of one extra wash with the RPE buffer and eluted in 50 *μ*L of nuclease free water and stored at −80°C.

### cDNA synthesis and analysis of plasma miRNA quality

First strand cDNA synthesis was performed from 1 *μ*L of RNA (in 10 *μ*L reactions) using the miRCURY universal cDNA synthesis kit per manufacturer instructions (Exiqon, Copenhagen, Denmark). The efficiency of RNA extraction was monitored by the addition of 2 fmol of a synthetic miRNA, UniSp2 (Exiqon), to the denatured plasma samples. The efficiency of the cDNA synthesis was monitored by the addition 0.15 fmol of a synthetic miRNA, UniSp6 (Exiqon), to the master mix of the reverse transcription reaction. The samples were run on a PTC-100 thermocycler (BioRad, Hercules, CA) for 60 min at 42°C, heat-inactivated for 5 min at 95°C, and then cooled to 4°C. All samples were stored at −20°C. The cDNA was diluted 1:40 in nuclease free water and real-time quantitative reverse transcription PCR (qPCR) was performed to assess the quality of the samples. Previously reported abundant endogenous miRNAs were detected along with the synthetic control miRNAs. Duplicate qPCR reactions were performed in a final volume of 10 *μ*L containing 5 *μ*L of PCR SYBR green master mix, 1 *μ*L of specific PCR primer (Exiqon), and 4 *μ*L of diluted cDNA template. Reactions were run on an ABI StepOnePlus real-time PCR system (Life Technologies, Carlsbad, CA) in 96-well optical plates. After a polymerase activation step at 95°C for 10 min, the samples were cycled 40 times at 95°C for 10 sec, 60°C for 1 min with ramp-rate of 1.6°C/sec. Melting curve analysis was performed on each reaction for quality control. Samples that had a single peak melting curve and Cq values of UniSp2 and UniSp6 within 1 standard deviation from the median were determined to have met quality control thresholds and chosen for further global analysis.

### Comprehensive profiling of plasma miRNAs in GEMM

For comprehensive miRNA analysis, qPCR was performed on samples from six wild-type, mice and 14 GEMM OS mice using Exiqon miRNome platform (mouse panels I+II, V2), which utilizes ready-to-use PCR panels and analyzes 752 miRNAs. Per manufacturer’s recommendations, the cDNA reaction was scaled up and 72 *μ*L of master mix and 8 *μ*L of RNA were combined and cDNA synthesis completed under the same conditions as above. The cDNA was diluted 55-fold in nuclease free water and combined in an equal volume of 2X SYBR green master mix and 10 *μ*L was added to each well (Exiqon). The samples were run on an LC480 instrument (Roche Applied Sciences) in 384-well optical plates. After polymerase activation at 95°C for 10 min, the samples were cycled 45 times at 95°C for 10 sec, 60°C for 1 min with a ramp-rate of 1.6°C/sec. Melting curve analysis was performed on each reaction for quality control. Raw data were loaded on GenEx Pro version 5.4.3.710 from MultiD (Göteborg, Sweden). After interplate calibration, data were tested for outliers with cutoff SD (cycles) = 0.25 and Grubbs test was performed with confidence level = 0.95. All outliers were deleted as recommended. Values above Cq > 37 were treated as background. All nonnumerical values were replaced and miRNAs with ≥75% missing values, both within biologic replicates and altogether, were removed from analysis. Missing values within biologic replicates were generated by imputing, whereas missing values were assigned a maximum of Cq = 38. The processed data were normalized to global mean of all remaining miRNAs. Data were converted to relative quantiles and log2 transformed for statistical testing. An unpaired two-tailed *t*-test was performed between the two different conditions and the *P*-values were corrected for multiple testing using Benjamini–Hochberg method. MicroRNAs with fold change ≥5 and *P*-value (BH-corrected) ≤0.005 were considered significant. Candidate miRNAs were chosen based on *P*-value of less than 0.005, sequence conservation in mice and humans, and scientific interest based on published literature. Additionally, GeNorm and NormFinder algorithms were run and identified miR-103, miR-191, and miR-423-3p as appropriate reference miRNAs 11.

### Validation of plasma miRNA levels in GEMM

A sample size calculation was performed for each candidate miRNA using a two tailed *t*-test with 90% power and an alpha error rate of 0.01. The largest sample size calculated was used (20 in each group). Candidate miRNAs were detected via qPCR under the same conditions as above on a StepOnePlus real-time PCR system (Life Technologies). Plasma miRNAs were analyzed in an independent set of 20 wild-type mouse samples and 20 GEMM samples (10 localized and 10 metastatic). All reactions were run in duplicate and each miRNA primer had at least one no template control. Three reference miRNAs, in addition to the spiked in UniSp2 were used for normalization in the validation experiments (ΔCq = NF − Cq_miRNA_) were NF is the normalization factor and NF = (Cq_miR-103_ + Cq_miR-191_ + Cq_miR-423-3p_ + Cq_UniSp2_)/4. All primers used in the murine and human studies were purchased from Exiqon (Catalog #203950, 204063, 204066, 204151, 204154, 204306, 204487, 204488, 204510). A two-sample, two-tailed Student’s *t*-test comparing the 2^−ΔCq^ values of the two groups was performed and a *P*-value of <0.05 was considered significant. Data were analyzed with SigmaPlot Software V10.0 and DataAssist Software V3.01 (Life Technologies).

### Mouse orthotopic OS transplantation, tumor monitoring, blood sampling

Athymic nude mice (*nu−/nu−*) were purchased from Jackson Laboratory (Bar Harbor, ME) at 42 days of age. A baseline blood sample was obtained via submandibular vein collection for each animal 12. Murine OS cells harvested during the exponential growth phase were injected intratibially at a concentration of 3 × 10^5^ cells/mouse mixed in Matrigel (BD Biosciences, San Jose, CA) under isoflurane anesthesia per IACUC protocols into 10 mice. Mice were monitored weekly for tumor formation and blood samples were taken at 2–5 week intervals to evaluate the levels of plasma miR-205-5p, miR-214, miR-335-5p, and miR-574-3p. Mice were euthanized when the tumor volume exceeded 10% of the body weight of the animal. A two-sample, two-tailed Student’s *t*-test comparing the 2^−ΔCq^ values of the baseline miRNA compared to each subsequent time point in each individual animal was performed and a *P*-value of <0.05 was considered significant.

### Doxorubicin treatment

Athymic mice were purchased as above and baseline blood samples were obtained on all animals. Ten mice underwent intratibial transplantation with murine OS cells as above and 10 mice underwent sham transplants with an intratibial injection of an equal volume saline and Matrigel (San Jose, CA). Mice were monitored biweekly for the tumor formation and mice were randomized into experimental groups when tumors were palpable. Thereafter, tumor volumes were measured biweekly with calipers and volumes calculated using Equation 1: *V* = *A* × *B*^2^/2 (*A* = largest diameter; *B* = smallest diameter) 13. Sham-transplanted mice were randomized 1:1 at the same time as the tumor bearing mice. The four experimental groups were as follows: (1) sham transplant/placebo treatment, (2) sham transplant/doxorubicin (DOX) treatment, (3) OS transplant/placebo treatment, and (4) OS transplant/DOX treatment. Mice received intraperitoneal DOX at 4 mg/kg diluted with isotonic phosphate-buffered saline (pH 7.4) to a final volume of 100 *μ*L/10 g of body weight weekly × 4 weeks 14,15. Mice in the placebo arms received intraperitoneal isotonic injections of 100 *μ*L/10 g of body weight. Blood samples were obtained weekly prior to each injection (DOX or placebo).

### Necropsy and evaluation of effect

Necropsy was completed and primary tumors were sent to the BCM histology core at the Breast Center for histologic confirmation of disease. Final tumor weight was determined after resection from each animal.

### Analysis of plasma miRNAs

Plasma preparation, RNA extraction, cDNA synthesis, and measurement of plasma miRNAs were performed by methods identical to those described in the GEMM samples. All samples at the time of randomization were considered one biologic group and the levels of miRNAs were compared to this group at weekly time points after randomization (up to 4 weeks of treatment).

### Human plasma samples

Human plasma samples were obtained from three sources. After approval from the BCM institutional review board, 16 OS patient plasma samples were obtained from The Texas Children’s Hospital Tissue Bank and 40 human plasma samples from OS patients were obtained through the Children’s Oncology Group (Protocol H-6650 and H-31361). An additional 11 OS samples were a kind gift from Dr. Chelouche Lev. Thirty healthy individual control plasma samples from 18 year-olds were purchased from Bioreclamation LLC (Hicksville, NY).

### Extraction of plasma RNA from human samples

Plasma preparation and RNA extraction are described above, with the exception that 250 *μ*L of human plasma was passed through a 0.22 *μ*m filter and a total volume of 200 *μ*L was used for RNA extraction. The organic extraction was up-scaled and 100 *μ*L of Qiazol reagent and 200 *μ*L of chloroform were used. RNA was extracted from the aqueous phase using the Qiagen miRNeasy kit as per manufacturer instructions and eluted in 50 *μ*L of nuclease free water.

### cDNA synthesis and analysis of plasma miRNA quality from human samples

First strand cDNA synthesis was performed from 2 *μ*L of RNA (in 10 *μ*L reactions) using the miRCURY universal cDNA synthesis kit per manufacturer instructions (Exiqon). The spike-in synthetic RNAs were used as above to monitor the efficiency of the RNA extraction and cDNA synthesis. The cDNA was diluted 1:20 in nuclease free water and qPCR was performed as above and samples that did not meet quality control thresholds were omitted.

### Comprehensive plasma miRNA expression profile to determine reference miRNAs

Comprehensive miRNA expression profiling was completed using the Exiqon miRNome platform (human panels I+II, V3) as above on 20 disease samples (10 localized, 10 metastatic) and 15 healthy controls to identify reference miRNAs in human OS plasma samples. The GeNorm algorithm identified miR-320a and miR-15a-5p as appropriate reference miRNAs.

### Validation of plasma miRNA levels in human samples

An independent set of 40 OS samples (Table1) and 30 healthy control samples was used to investigate the four miRNAs validated in the GEMM samples. One sample did not meet quality control thresholds and was omitted from final analysis. All reactions were run in triplicate and each miRNA primer had at least one no template control. Both reference miRNAs, in addition to the spiked in UniSp2 were used for normalization in the validation experiments (ΔCq = NF − Cq _miRNA_) were NF is the normalization factor and NF = (Cq_miR-320a_ + Cq_miR-15a-5p_ + Cq_UniSp2_)/3. A two-sample, two-tailed Student’s *t*-test comparing the 2^−ΔCq^ values of the two groups was performed and a *P*-value of <0.05 was considered significant.

**Table 1:** Clinical demographics for the OS patients from whom plasma samples were obtained

Patient	Sex	Age (years)	Disease at diagnosis	Tumor site	Follow-up time (years)
1	Male	8.1	Localized	Femur	2.9
2	Male	21.3	Localized	Scapula	0.8
3	Female	17.8	Localized	Femur	2.1
4	Male	16.3	Localized	Femur	1.0
5	Male	13.1	Localized	Femur	1.3
6	Male	9.3	Localized	Tibia	2.8
7	Female	14.2	Localized	Tibia	2.1
8	Male	12.2	Localized	Femur	2.0
9	Female	15.9	Localized	Femur	2.1
10	Female	11.9	Localized	Femur	2.0
11	Male	12.1	Localized	Femur	1.9
12	Male	16.6	Localized	Humerus	2.3
13	Female	7.5	Localized	Femur	0.7
14	Female	12.1	Localized	Tibia	2.5
15	Female	16.9	Localized	Humerus	2.0
16	Male	19.1	Localized	Humerus	2.0
17	Female	16.2	Localized	Tibia	2.0
18	Female	17.1	Localized	Femur	2.4
19	Male	13.9	Localized	Femur	1.1
20	Male	22.7	Metastatic	Tibia	1.0
21	Female	9.0	Metastatic	Femur	1.3
22	Male	10.0	Metastatic	Femur	1.2
23	Female	14.3	Metastatic	Humerus	1.0
24	Male	12.7	Metastatic	Femur	0.7
25	Male	8.2	Metastatic	Other	2.2
26	Male	14.9	Metastatic	Femur	2.0
27	Female	16.4	Metastatic	Femur	1.9
28	Female	17.1	Metastatic	Femur	1.6
29	Male	12.7	Metastatic	Tibia	1.9
30	Male	4.4	Metastatic	Tibia	2.0
31	Female	17.3	Metastatic	Scapula	2.0
32	Male	11.4	Metastatic	Femur	2.0
33	Male	14.5	Metastatic	Femur	0.9
34	Male	15.5	Metastatic	Femur	1.0
35	Male	13.2	Metastatic	Femur	0.7
36	Male	11.6	Metastatic	Femur	0.5
37	Female	5.6	Metastatic	Femur	1.0
38	Female	7.7	Metastatic	Femur	1.2
39	Female	12.3	Metastatic	Tibia	1.0

## Results

### Identification and validation of plasma OS-associated miRNAs in GEMM

**Figure 1:**
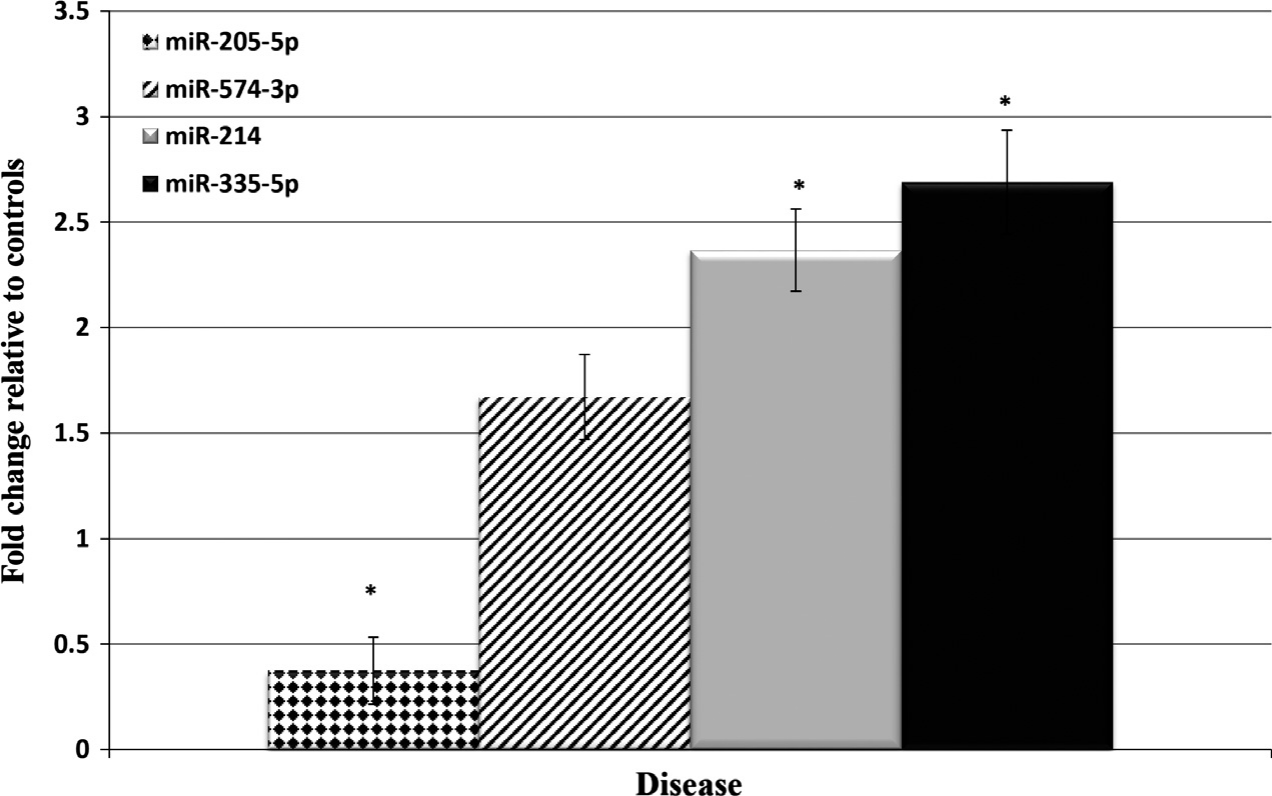
Plasma microRNA expression profile in GEMM samples. qPCR analysis of plasma microRNAs from control and mice afflicted with OS. Analysis of miR-205-5p, -574-3p, -214 and -335-5p were performed in mice with OS compared to age matched wild-type litter mate controls. Statistically significant differences between groups were assessed using a two-sample, two-tailed Student’s *t*-test comparing the 2^−ΔCt^ values of the two groups. (*P < 0.05).

In order to identify noninvasive novel biomarkers for OS, we measured the levels of 752 unique plasma miRNAs in two biologic groups, one being GEMM mice with OS 8 and the other age matched wild-type control litter mates. Principal component analysis demonstrated variation in plasma miRNA expression profiles from animal to animal, but the biologic groups clustered together, demonstrating expression profiles were most similar within each biologic group (Fig. S1). Besides the identification of differential expression of miRNAs, our initial studies identified three reference endogenous miRNAs (miR-103, miR-191, and miR-423-3p). These were identified with GeNorm and Normfinder, which are mathematical models of gene expression and provide a direct measure for the estimated expression variation (Fig. S2) 11. We chose endogenous controls that the two algorithms agreed upon and were not cellular in nature (i.e., RNU6). Four miRNAs (miR-205-5p-5p, miR-214, miR-335-5p, and miR-574-3p) were chosen as candidate miRNAs based on reports in published literature, the presence of a conserved known human homologue, and the fold change in the global qPCR analysis. Three of the four miRNAs (miR-205-5p-5p, miR-214, and miR-335-5p) were validated in an independent set of diseased and wild-type mice to be statistically significant (*P* < 0.05) using a two-sample, two-tailed Student’s *t*-test comparing the 2^−ΔCq^ values of the two groups MicroRNA-574-3p was not statistically significant in final statistical analysis, but was included in simultaneous studies based on preliminary results (*P *=* *0.15) (Fig.1). MicroRNA 205-5p was decreased 2.68-fold (95% CI 2.17–2.89) in diseased mice compared to controls, whereas miR-214 and miR-335-5p were increased 2.37- (95% CI 1.81–2.93) and 2.69-fold (95% CI 2.12–3.26), respectively, in diseased mice.

### Analysis of miRNA profile during tumor formation in an immunodeficient allograft mouse model

**Figure 2:**
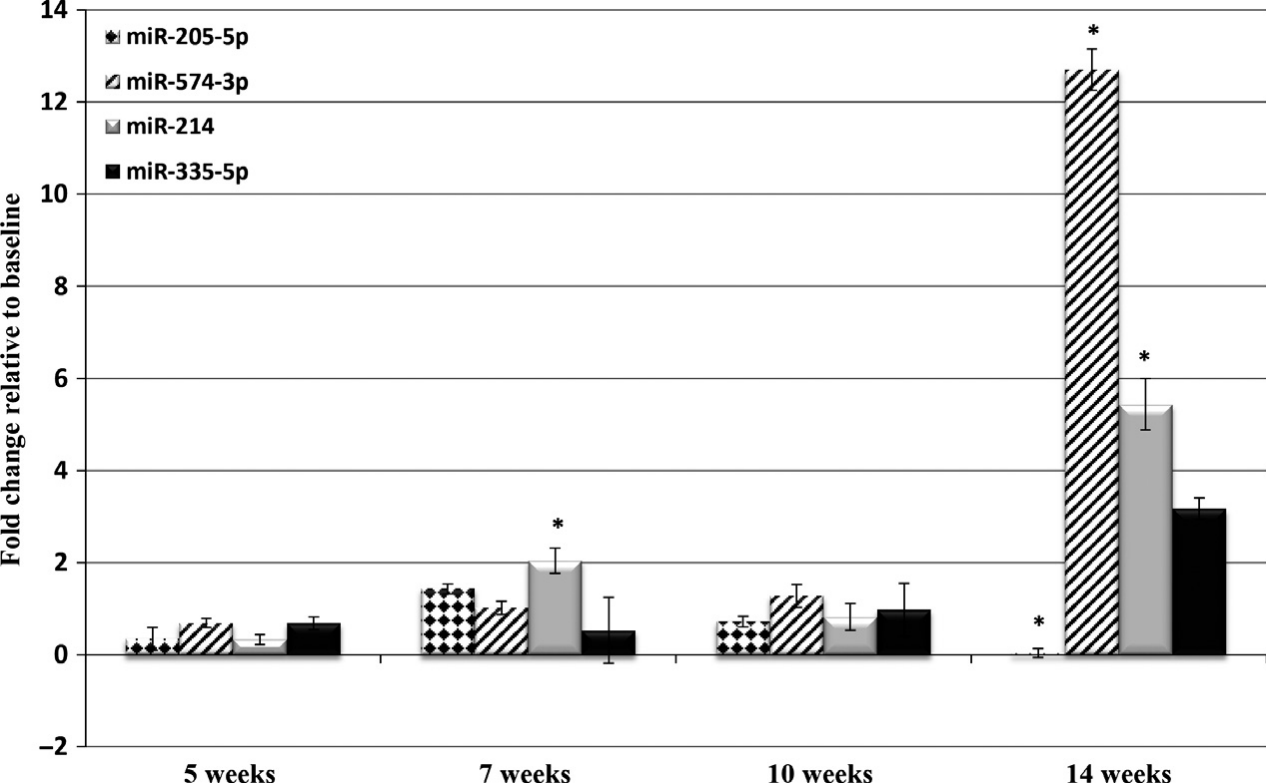
Plasma microRNA expression in an orthotopic transplanted animal. The animal developed OS 14 weeks after orthotopic transplantation with murine OS cells. The levels of miR-205-5p, miR-574-3p, and miR-214 were significant from baseline at the time of tumor development (14 week time point). Statistically significant differences were assessed using a two-sample, two-tailed Student’s *t*-test comparing the 2^−ΔCt^ values of the two groups. (*P < 0.05).

After determination of a signature plasma miRNA profile capable of delineating healthy from disease state, we were interested in analyzing the use of these plasma miRNAs as prospective markers for disease development and progression. Therefore, we monitored the levels of miR-205-5p, miR-214, miR-335-5p, and miR-574-3p prior to and serially after transplantation of OS cells. Nine of 10 mice injected with OS cells developed tumors and the levels of each miRNA in one animal are shown in Figure2. This animal showed no signs of tumor formation until 14 weeks after injection, at which time it was euthanized and OS was confirmed on histology. At 14 weeks from injection and in the presence of tumor, miR-205-5p was significantly downregulated and miR-214 and miR-574-3p were upregulated. The graphs for the remaining eight mice can be found in Figure S3–S4. These results demonstrate the potential clinical use of plasma miRNAs to assess disease development.

### Analysis of changes in the levels of plasma miRNA after doxorubicin treatment

**Figure 3:**
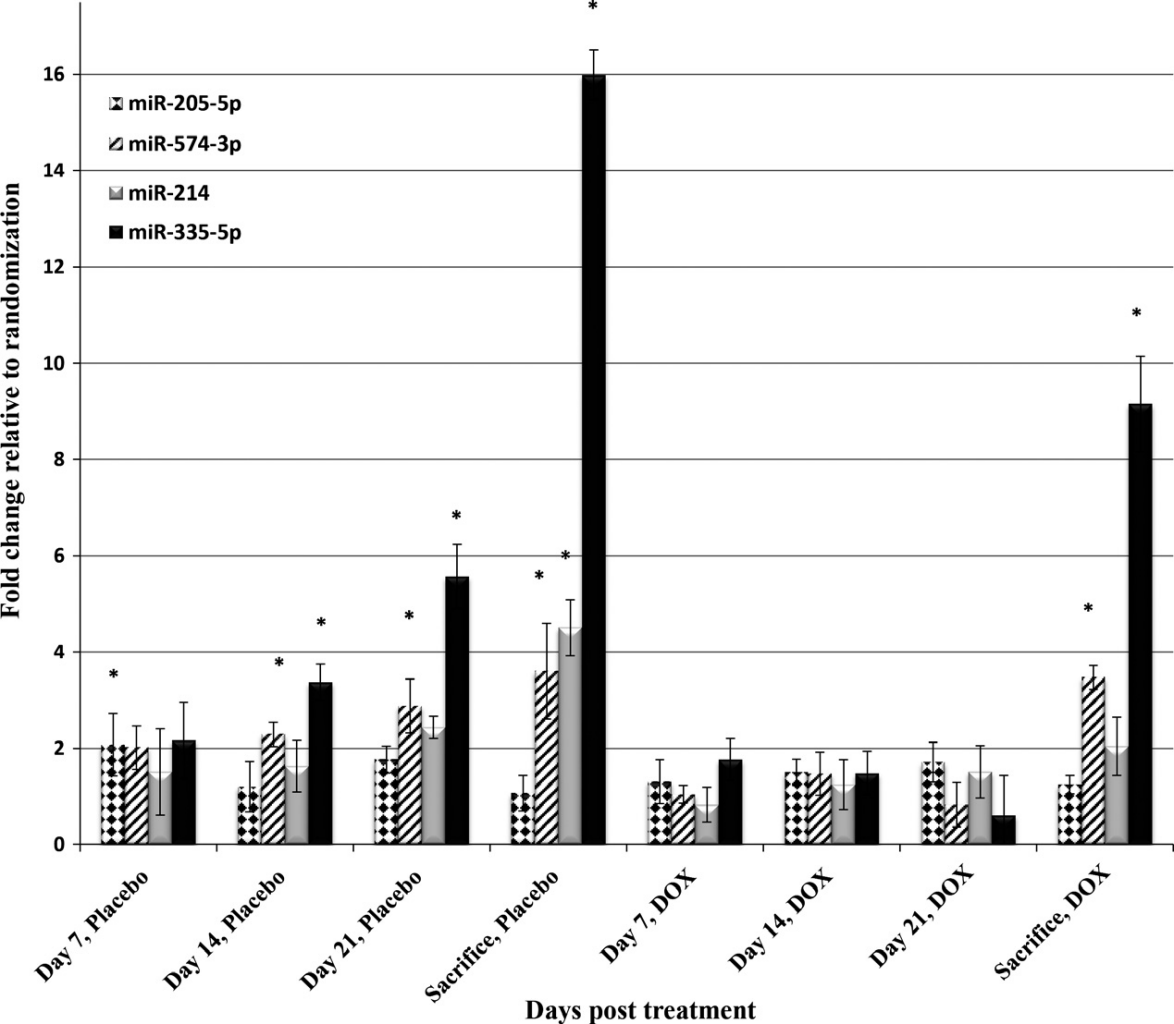
Plasma microRNA expression assessing chemotherapy responsiveness in orthotopic OS model. Mice were injected intratibially with mouse OS cells and then randomized to receive placebo or doxorubicin. Statistically significant differences were assessed using a two-sample, two-tailed Student’s *t*-test comparing the 2^−ΔCt^ values in each group compared to microRNA levels at randomization. (*P < 0.05).

We next explored the potential for a plasma miRNA signature to be used as a real-time marker of therapeutic responsiveness. To investigate this question, we performed prospective analysis of placebo and chemotherapy-treated orthotopic models of murine OS. All transplanted mice (*n* = 10) developed OS tumors and mice treated with DOX tolerated the chemotherapy treatment with no significant morbidities (Fig. S5A), but had statistically significant (*P *<* *0.05) growth inhibition of the tumor when compared with placebo-treated mice (*P* = 0.017, Fig. S5B and C). As shown in Figure3, three biologic groups were compared pairwise. The first group included all mice (*n* = 10) at the time of tumor formation and immediately prior to randomization. This group was used as the comparator for the remaining analyses. After treatment started (placebo or DOX), the levels of each miRNA were measured weekly in all animals and then the mean value for each treatment group (*n* = 5 DOX, *n* = 5 placebo) was compared to the mean value at randomization, using a two-sample, two-tailed, Student’s *t*-test comparing the 2^−ΔCq^ values between groups. The miRNA pattern of expression remained similar to previous experiments with increased levels of mir-214, miR-335-5p, and miR-574-3p in the diseased state. Furthermore, the levels of the miR-214, miR-335-5p, and miR-574-3p increased significantly at earlier time points in the placebo-treated animals compared to those animals treated with DOX. The animals treated with DOX only reached statistical significance at the time of sacrifice. There was no statistical significance when comparing the expression levels in the placebo versus DOX groups, which is likely because both groups had active growing tumors. We also performed parallel analysis of sham transplanted mice that were randomized to placebo or DOX treatment and there were no significant miRNA level changes seen in either group (Fig. S6).

### Validation in OS-associated plasma miRNAs in human samples

After utilizing our GEMM to identify a novel plasma miRNA profile, we assessed the expression of the miRNA profile in human OS patient plasma samples. However, initially it was essential to determine endogenous reference miRNAs that could be used for normalization of our samples. Through the comprehensive, global analysis of 20 OS and 15 healthy human samples we identified two reference miRNAs for plasma miRNAs in human OS patients. GeNorm identified miR-15a-5p and miR-320a as suitable reference miRNAs with standard deviations of 0.126 and 0.148, respectively.

**Figure 4:**
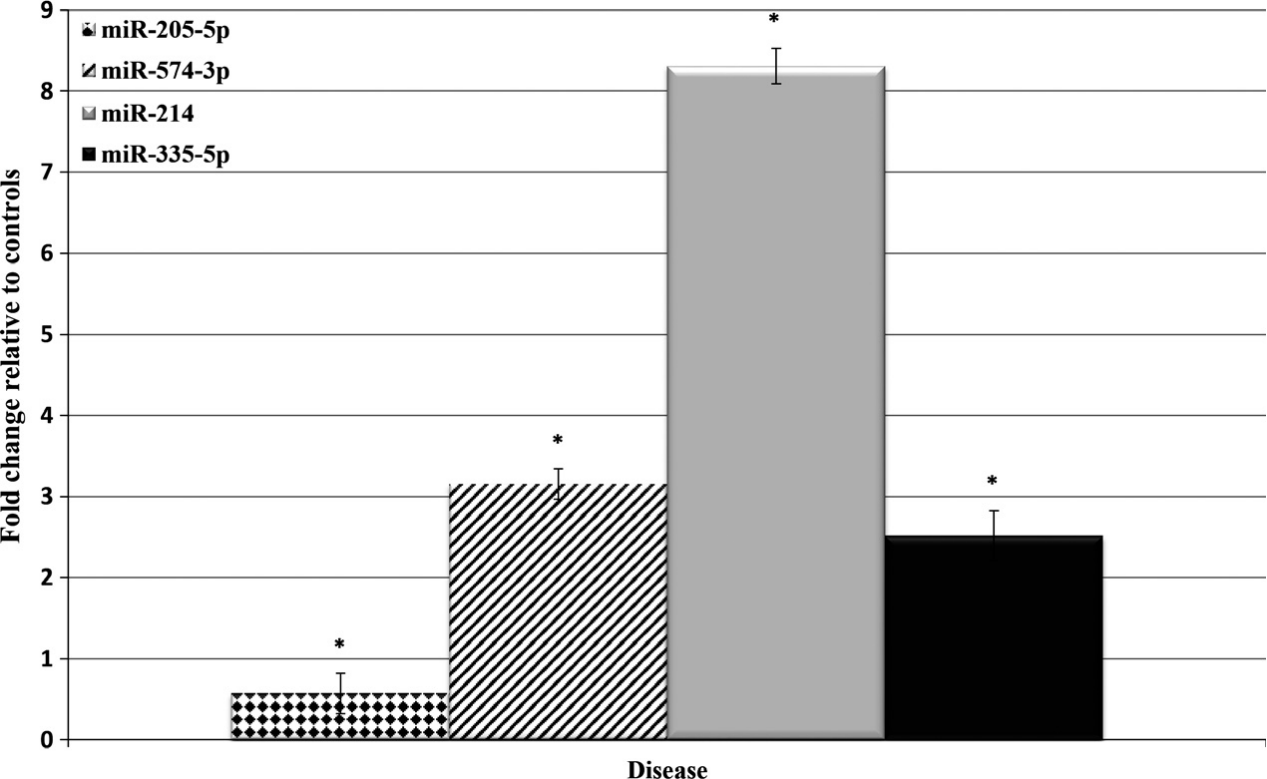
Plasma microRNA expression in human OS patient plasma. qPCR analysis of plasma microRNAs from healthy control and OS patient plasma samples. Statistically significant differences between groups were assessed using a two-sample, two-tailed Student’s *t*-test comparing the 2^−ΔCt^ values of the two groups. (*P < 0.05).

**Figure 5:**
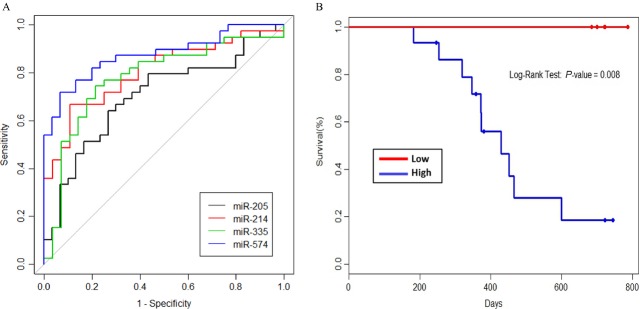
Receiver operating characteristic (ROC) curve analysis of plasma miRNAs. (A) ROC curves were constructed for individual miRNAS. Areas under the curve (AUCs) were 0.70 (95% CI 0.576–0.827), 0.80 (95% CI 0.699–0.909), 0.78 (95% CI 0.661–0.898), and 0.88 (95% CI 0.794–0.957) for miR-205-5p, miR-214, miR-335-5p, and miR-574-3p, respectively. (B) Kaplan–Meier curve for metastatic patients based upon plasma miR-214 levels at time of diagnosis.

Subsequently, we measured the levels of plasma miR-205-5p, miR-214, miR-335-5p, and miR-574-3p by qPCR in an independent set of 40 children with OS and 30 healthy controls. All four candidate miRNAs were statistically significant (*P *<* *0.05) using a two-sample, two-tailed Student’s *t*-test comparing the 2^−ΔCq^ values of the control and disease groups in an independent set of human samples (Fig.4). Receiver operating characteristics curve analyses were conducted to estimate optimal cut-points for the four plasma miRNAs to discriminate OS patients from healthy controls. As shown in Figure5A, the areas under the curves (AUCs) were 0.70 (95% CI 0.576–0.827), 0.8 0(95% CI 0.699–0.909), 0.78 (95% CI 0.661–0.898), and 0.88 (95% CI 0.794–0.957) for miR-205-5p, miR-214, miR-335-5p, and miR-574-3p, respectively.

Optimal cut-points from the ΔCq values were determined for each miRNA using the Youden index. A value falling above or below (depending on direction of change in the diseased population) should identify diseased patients from healthy patients. The ΔCq cut-points were 8.34 for miR-205-5p, 10.31 for miR-214, 9.78 for miR-335-5p and 6.08 for miR-574-3p. Additionally, we investigated whether these cut-points could be used as a prognostic marker for outcome. We found that miR-214 levels differ significantly between metastatic and nonmetastatic patients (*P* = 0.016), suggesting an association between metastatic status and miR-214 levels. Next, we further stratified all patients that presented with metastatic disease and noted that low plasma miR-214 levels could distinguish a subpopulation of patients with significantly enhanced overall survival (*P* = 0.008), Figure5B.

## Discussion

The study of miRNAs as biomarkers in acellular body fluids such as plasma has expanded rapidly in the last several years, but continues to be complicated by several preanalytic and technical factors that require consideration during investigational studies. First, when carrier RNA is used to reduce the loss of the miRNA subpopulation, efforts to assess the quality and quantity of the extracted RNA through traditional methods such as spectrophotometry and automated capillary electrophoresis instruments are inaccurate and standardization of RNA input quantity is impossible. However, if no carrier RNA is used, the miRNA subpopulation may be too small to accurately quantify. Second, due to the acellular nature of plasma and serum, typical reference miRNAs used for tissue analysis (i.e., RNU6) cannot be used for normalization. To compound the problem, it appears that each disease population has a unique set of endogenous reference miRNAs and the reference miRNAs do not translate from one population to another (i.e., breast cancer to colon cancer) 16.

Through pilot large-scale comprehensive profiling studies, we identified three reference miRNAs, miR-109, miR-191, and miR-423-3p with GeNorm and Normfinder that were invariant in the mouse samples and were used as reference miRNAs for those studies. Similarly, two reference miRNAs, miR-320a and miR-15a-5p were identified by GeNorm and used as endogenous reference miRNAs for human OS studies utilizing plasma. We believe the identification of these reference miRNAs allowed for accurate normalization and relative quantification of the experimental plasma miRNA levels for these and future studies.

Our studies revealed plasma miR-205-5p was downregulated in GEMM mice with OS compared to wild-type littermate controls, whereas levels of miR-214 and miR-335-5p were significantly higher in GEMM mice. The level of plasma miR-574-3p was not statistically significant in the GEMM studies, however, based on the results from the DOX experiments that were being performed simultaneously with the validation set; we decided to further investigate miR-574-3p in the human samples.

Besides our ability to identify a miRNA signature that can distinguish healthy from diseased patients, our investigations also implied that these signatures have the potential to monitor chemotherapeutic responsiveness, but additional studies need to be carried out to validate these results. In the DOX experiments, the magnitude of the alteration in the levels of plasma miR-205-5p, miR-214, miR-335-5p, and miR-574-3p was less in the DOX-treated mice compared to those mice treated with placebo. Furthermore, the mice treated with DOX took several weeks longer to achieve significant changes from baseline compared to those mice treated with placebo. Although the differences between the DOX group and the placebo group were not statistically significant, these results suggest that the miRNA profile may be useful as a noninvasive marker to monitor the tumor response to chemotherapy. These findings need to be evaluated prospectively in human samples where complete remission is achieved and corroboration with other measures (e.g., radiographic, histologic) of disease response. In addition, studies assessing the correlation of plasma miRNA alterations with changes in tumor cell miRNA levels are still warranted.

The levels of all four miRNAs investigated in the GEMM were statistically significant in the human samples with decreased levels of miR-205-5p and elevated levels of miR-214, miR-335-5p, and miR-574-3p in OS patients. Our studies are a first of its kind in translating mouse model findings to human patient samples and our results suggest that cross-species validation of highly conserved miRNAs can be achieved using appropriate model systems. This concept is extremely important for rare tumors such as OS where clinically annotated samples are scarce and difficult to obtain.

Furthermore, in a training set of human patient samples, receiver operating characteristic curves showed that the four miRNAs have sufficient sensitivity and specificity to identify those patients with OS from healthy controls. The cut-points determined in the training set need to be validated in an independent set of human patient samples to fully complete the establishment of this signature profile in OS.

Metastatic disease at diagnosis is the best predictor of outcome in OS and only approximately 20% of metastatic patients will survive. However, presently we do not have the ability to immediately identify that subpopulation of metastatic patients that will have sustained response to therapy and survive. Analysis of our metastatic cohort suggests that plasma miR-214 levels can identify this subpopulation, as the 20% of metastatic patients with low miR-214 levels are all presently alive. This result needs to be repeated in a larger sample size with longer follow-up times, but plasma miR-214 could be the first biomarker identified that can discriminate which metastatic patients will have a good outcome with current therapeutic regimens.

There is one previous report investigating specific circulating miRNAs in OS 17. Our study differs from that report in several critical ways that add to the current understanding of circulating miRNAs in OS. First, we completed a comprehensive screen of 752 plasma miRNAs in the GEMM. This comprehensive screen did not restrict our candidate miRNAs to those that had been previously reported as altered in cell lines or tumor samples. Second, the use of the endogenous reference miRNAs and the spiked in miRNA for normalization in both the GEMM and the human samples adequately addresses the biologic variability that is inherent in these samples. While our study is the first report of plasma miR-205-5p, miR-214, miR-335-5p, and miR-574-3p to be used as biomarkers, the literature supports that each of these miRNAs may have an important biologic function in OS.

MicroRNA 205-5p has been reported to act as a tumor suppressor in several cancer types including breast cancer and prostate cancer 18. Furthermore, recent evidence suggests that miR-205-5p is highly specific to epithelium, which may explain the results in the DOX experiments which do not show a significant decrease in the disease state compared to baseline likely due to the method of blood sampling. In the initial screen, blood was obtained via cardiac puncture and in the DOX experiments blood was obtained via submandibular vein sampling and thus the submandibular samples will have more epithelial contamination compared to cardiac puncture. This highlights the importance of standardizing the preanalytic variables affecting results when utilizing miRNAs as noninvasive biomarkers.

Data are now emerging on the importance of miR-214 in OS. Upregulated expression of miR-214 in tumors is linked to tumor progression and poor prognosis in OS and a proposed mechanism of action is that miR-214 promotes OS proliferation and invasion through direct suppression of leucine zipper, putative tumor suppressor 1 (LZTS1) 19,20. These reports corroborate our data that plasma miR-214 is associated with metastatic disease and can be used as both a diagnostic biomarker and a prognostic biomarker for patient outcome. Based on this data, further investigations in miR-214 as it relates to OS are warranted.

Both miR-335-5p and miR-574-3p have been reported to be important in bone and cartilage development and differentiation through the regulation of the Wnt pathway and *SOX9* expression, respectively 21,22. Previous reports have shown activation of the Wnt signaling pathway may contribute to OS development and progression, however, additional studies are required to delineate the critical components and their precise functions within OS 23. Finally, miR-574-3p has recently been reported to play an important role in maintaining mesenchymal stem cell multipotency, which is of interest for OS, as it is thought that the mesenchymal stem cell is the cell of origin in OS 22.

In summary, we utilized a GEMM of OS to discover and validate a signature profile of plasma miRNAs that can distinguish OS from healthy animals in retrospective and prospective studies, and potentially monitor therapeutic responsiveness. We were able to translate these findings to an independent set of 40 human plasma samples. Using receiver operating characteristic curves, we showed that each miRNA has sufficient specificity and sensitivity to detect OS in human plasma samples. Lastly, we have identified that low plasma levels of miR-214 in metastatic patients at time of diagnosis is associated with an excellent prognosis.

We believe that the identification of the miRNA signature within our report enhances the understanding of OS, and the technical approaches we have established to evaluate reference and experimental miRNAs are a tremendous advancement in the field of circulating miRNAs for OS that could be applied to other diseases.

## Conflict of Interest

None declared.

## Supporting Information

**Figure S1.** Principal component analysis of miRNA expression in diseased and control mice demonstrate expression profiles are most similar within biologic groups. **Figure S2.** *M* values and stability factors for GeNorm and NormFinder identify miR-423-3p, miR-191, and miR-103 as endogenous reference miRNAs for mouse experiments. **Figure S3.** Plasma microRNA expression in an orthotopic transplanted animals with localized disease. Graphs A–C each represent an individual animal. All animals developed tumors at 14 weeks and comparisons of miRNA levels were made to respective baseline levels in each animal prior to transplantation with OS cells. Statistically significant differences were assessed using a two-sample, two-tailed Student’s *t*-test comparing the 2^−ΔCt^ values of the two groups. (**P* < 0.05). **Figure S4.** Plasma microRNA expression in an orthotopic transplanted animals with metastatic disease. Graphs A–E each represent an individual animal. All animals developed tumors at 7 weeks and comparisons of miRNA levels were made to respective baseline levels in each animal prior to transplantation with OS cells. Statistically significant differences were assessed using a two-sample, two-tailed Student’s *t*-test comparing the 2^−ΔCt^ values of the two groups. (**P* < 0.05). **Figure S5.** Orthotopic transplantation mouse and tumor volume parameters. (A) No significant difference in mouse weights during chemotherapy treatment. (B) Tumor volume was significantly larger in placebo-treated mice after 28 days of treatment (C) Tumor weight was significantly higher at necropsy in placebo-treated group than in doxorubicin-treated group. Analyses performed using a two sampled, two-tailed Student’s *t*-test with **P* < 0.05. **Figure S6.** Plasma microRNA expression after sham transplantation. qPCR analysis of miR-205-5p, -574-3p, -214 and -335-5p upon randomization and at time of sacrifice in placebo treated and DOX treated. No significant changes were noted from randomization in the expression of any of the miRNAs in either the placebo- or doxorubicin-treated mice when sham transplants were performed. Analyses performed using a two sampled, two-tailed Student’s *t*-test with **P* < 0.05.

## References

[b1] Mirabello L, Troisi RJ, Savage SA (2009). Osteosarcoma incidence and survival rates from 1973 to 2004: data from the surveillance, epidemiology, and end results program. Cancer.

[b2] Calin GA, Croce CM (2006). MicroRNA signatures in human cancers. Nat. Rev. Cancer.

[b3] Cortez MA, Ivan C, Zhou P, Wu X, Ivan M, Calin GA (2010). microRNAs in cancer: from bench to bedside. Adv. Cancer Res.

[b4] Cortez MA, Bueso-Ramos C, Ferdin J, Lopez-Berestein G, Sood AK, Calin GA (2011). MicroRNAs in body fluids–the mix of hormones and biomarkers. Nat. Rev. Clin. Oncol.

[b5] Kosaka N, Iguchi H, Ochiya T (2010). Circulating microRNA in body fluid: a new potential biomarker for cancer diagnosis and prognosis. Cancer Sci.

[b6] Ng EK, Chong WW, Jin H, Lam EK, Shin VY, Yu J (2009). Differential expression of microRNAs in plasma of patients with colorectal cancer: a potential marker for colorectal cancer screening. Gut.

[b7] Selth LA, Townley S, Gillis JL, Ochnik AM, Murti K, Macfarlane RJ (2011). Discovery of circulating microRNAs associated with human prostate cancer using a mouse model of disease. Int. J. Cancer.

[b8] Zhao S, Kurenbekova L, Gao Y, Roos A, Creighton CJ, Rao P (2015). NKD2, a negative regulator of Wnt signaling, suppresses tumor growth and metastasis in osteosarcoma. Oncogene.

[b9] Cochrane RL, Clark SH, Harris A, Kream BE (2007). Rearrangement of a conditional allele regardless of inheritance of a Cre recombinase transgene. Genesis.

[b10] Dumble ML, Donehower LA, Lu X (2003). Generation and characterization of p53 mutant mice. Methods Mol. Biol.

[b11] Andersen CL, Jensen JL, Orntoft TF (2004). Normalization of real-time quantitative reverse transcription-PCR data: a model-based variance estimation approach to identify genes suited for normalization, applied to bladder and colon cancer data sets. Cancer Res.

[b12] Golde WT, Gollobin P, Rodriguez LL (2005). A rapid, simple, and humane method for submandibular bleeding of mice using a lancet. Lab Anim. (NY).

[b13] Yao C, Wu S, Li D, Ding H, Wang Z, Yang Y (2012). Co-administration phenoxodiol with doxorubicin synergistically inhibit the activity of sphingosine kinase-1 (SphK1), a potential oncogene of osteosarcoma, to suppress osteosarcoma cell growth both in vivo and in vitro. Mol. Oncol.

[b14] Kaminskas LM, McLeod VM, Kelly BD, Cullinane C, Sberna G, Williamson M (2012). Doxorubicin-conjugated PEGylated dendrimers show similar tumoricidal activity but lower systemic toxicity when compared to PEGylated liposome and solution formulations in mouse and rat tumor models. Mol. Pharm.

[b15] Keith WN, Mee PJ, Brown R (1990). Response of mouse skin tumors to doxorubicin is dependent on carcinogen exposure. Cancer Res.

[b16] Pritchard CC, Cheng HH, Tewari M (2012). MicroRNA profiling: approaches and considerations. Nat. Rev. Genet.

[b17] Ouyang L, Liu P, Yang S, Ye S, Xu W, Liu X (2013). A three-plasma miRNA signature serves as novel biomarkers for osteosarcoma. Med. Oncol.

[b18] Qin AY, Zhang XW, Liu L, Yu JP, Li H, Wang SZ (2013). MiR-205 in cancer: an angel or a devil?. Eur. J. Cell Biol.

[b19] Xu Z, Wang T (2014). miR-214 promotes the proliferation and invasion of osteosarcoma cells through direct suppression of LZTS1. Biochem. Biophys. Res. Commun.

[b20] Wang Z, Cai H, Lin L, Tang M, Cai H (2014). Upregulated expression of microRNA-214 is linked to tumor progression and adverse prognosis in pediatric osteosarcoma. Pediatr. Blood Cancer.

[b21] Zhang J, Tu Q, Bonewald LF, He X, Stein G, Lian J (2011). Effects of miR-335-5p in modulating osteogenic differentiation by specifically downregulating Wnt antagonist DKK1. J. Bone Miner. Res.

[b22] Guerit D, Philipot D, Chuchana P, Toupet K, Brondello JM, Mathieu M (2013). Sox9-regulated miRNA-574-3p inhibits chondrogenic differentiation of mesenchymal stem cells. PLoS ONE.

[b23] Cai Y, Cai T, Chen Y (2014). Wnt pathway in osteosarcoma, from oncogenic to therapeutic. J. Cell. Biochem.

