# Supplementary material for: Cross-species identification of a plasma microRNA signature for detection, therapeutic monitoring, and prognosis in osteosarcoma
Source: Cancer Med. 2015 Mar 17;4(7):977–88. doi: 10.1002/cam4.438 (PMC4529336; doi:10.1002/cam4.438)
Supplement: Supplementary file 1 [file cam40004-0977-sd1.docx]

**Supporting Information Legends**

**Supporting Information Figure 1.** Principal component analysis of miRNA expression in diseased and control mice demonstrate expression profiles are most similar within biologic groups.

**Supporting Information Figure 2.** M values and stability factors for GeNorm and NormFinder identify miR-423-3p, miR-191, and miR-103 as endogenous reference miRNAs for mouse experiments.

**Supporting Information Figure 3.** Plasma microRNA expression in an orthotopic transplanted animals with localized disease. Graphs A-C each represent an individual animal. All animals developed tumors at 14 weeks and comparisons of miRNA levels were made to respective baseline levels in each animal prior to transplantation with OS cells. Statistically significant differences were assessed using a two-sample, two-tailed Student’s t-test comparing the 2^-ΔCt^ values of the two groups. (*p < 0.05).

**Supporting Information Figure 4.** Plasma microRNA expression in an orthotopic transplanted animals with metastatic disease. Graphs A-E each represent an individual animal. All animals developed tumors at 7 weeks and comparisons of miRNA levels were made to respective baseline levels in each animal prior to transplantation with OS cells. Statistically significant differences were assessed using a two-sample, two-tailed Student’s t-test comparing the 2^-ΔCt^ values of the two groups. (*p < 0.05).

**Supporting Information Figure 5.** Orthotopic transplantation mouse and tumor volume parameters. (A) No significant difference in mouse weights during chemotherapy treatment. (B) Tumor volume was significantly larger in placebo treated mice after 28 days of treatment (C) Tumor weight was significantly higher at necropsy in placebo treated group than in doxorubicin treated group. Analyses done using a two sampled, two tailed Student’s t-test with *p<0.05.

**Supporting Information Figure 6.** Plasma microRNA expression after sham transplantation. qPCR analysis of miR-205-5p, -574-3p, -214 and -335-5p upon randomization and at time of sacrifice in placebo treated and DOX treated. No significant changes were noted from randomization in the expression of any of the miRNAs in either the placebo or doxorubicin treated mice when sham transplants were performed. Analyses done using a two sampled, two tailed Student’s t-test with *p<0.05.
