# Supplementary figures and images for: Cross-species identification of a plasma microRNA signature for detection, therapeutic monitoring, and prognosis in osteosarcoma
Source: Cancer Med. 2015 Mar 17;4(7):977–88. doi: 10.1002/cam4.438 (PMC4529336; doi:10.1002/cam4.438)

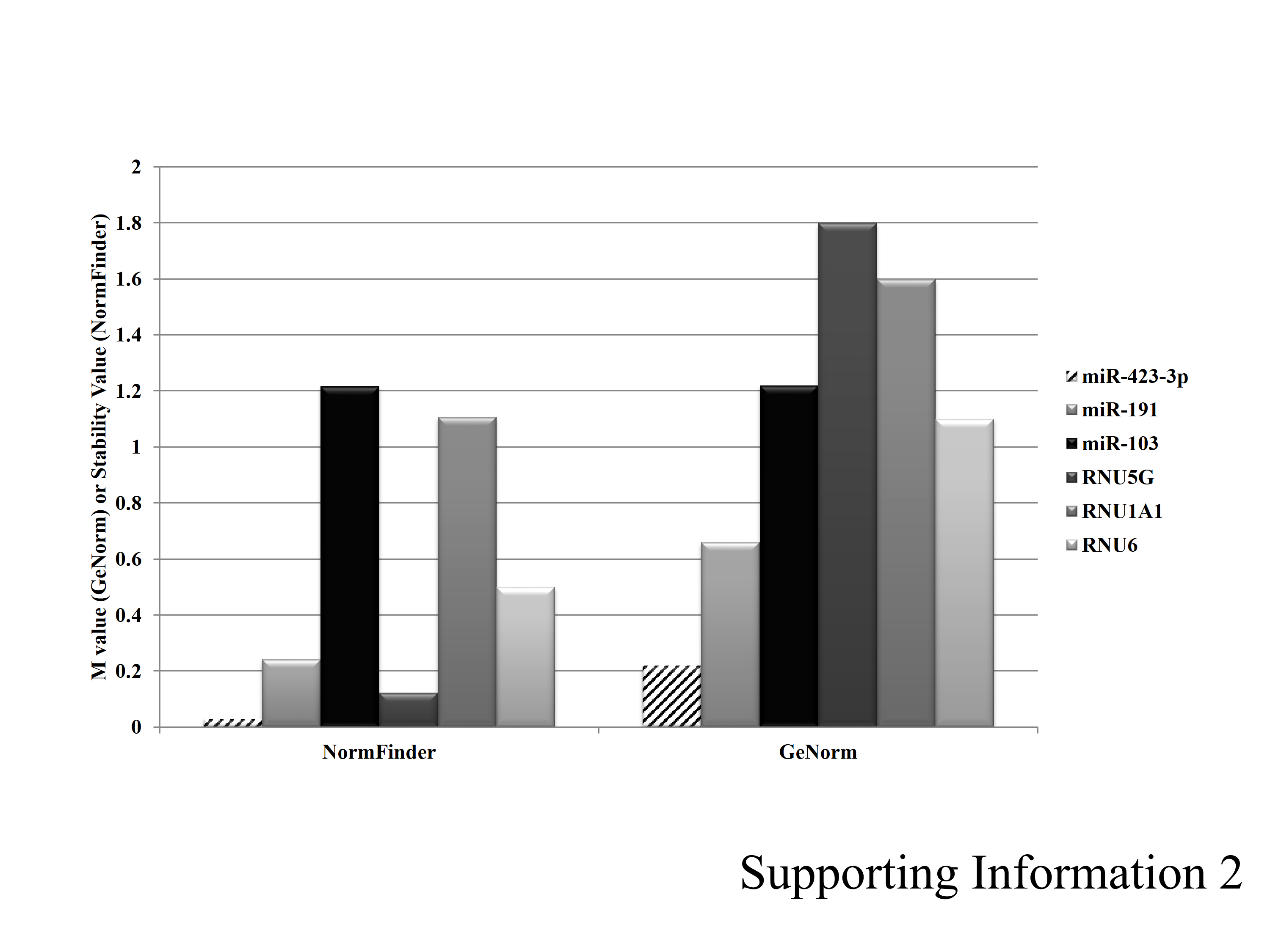

Supplement: Supplementary file 2 [file cam40004-0977-sd2.zip › CAM4_438_SUPP0002-Supp Info 2 FinalHR.tif]

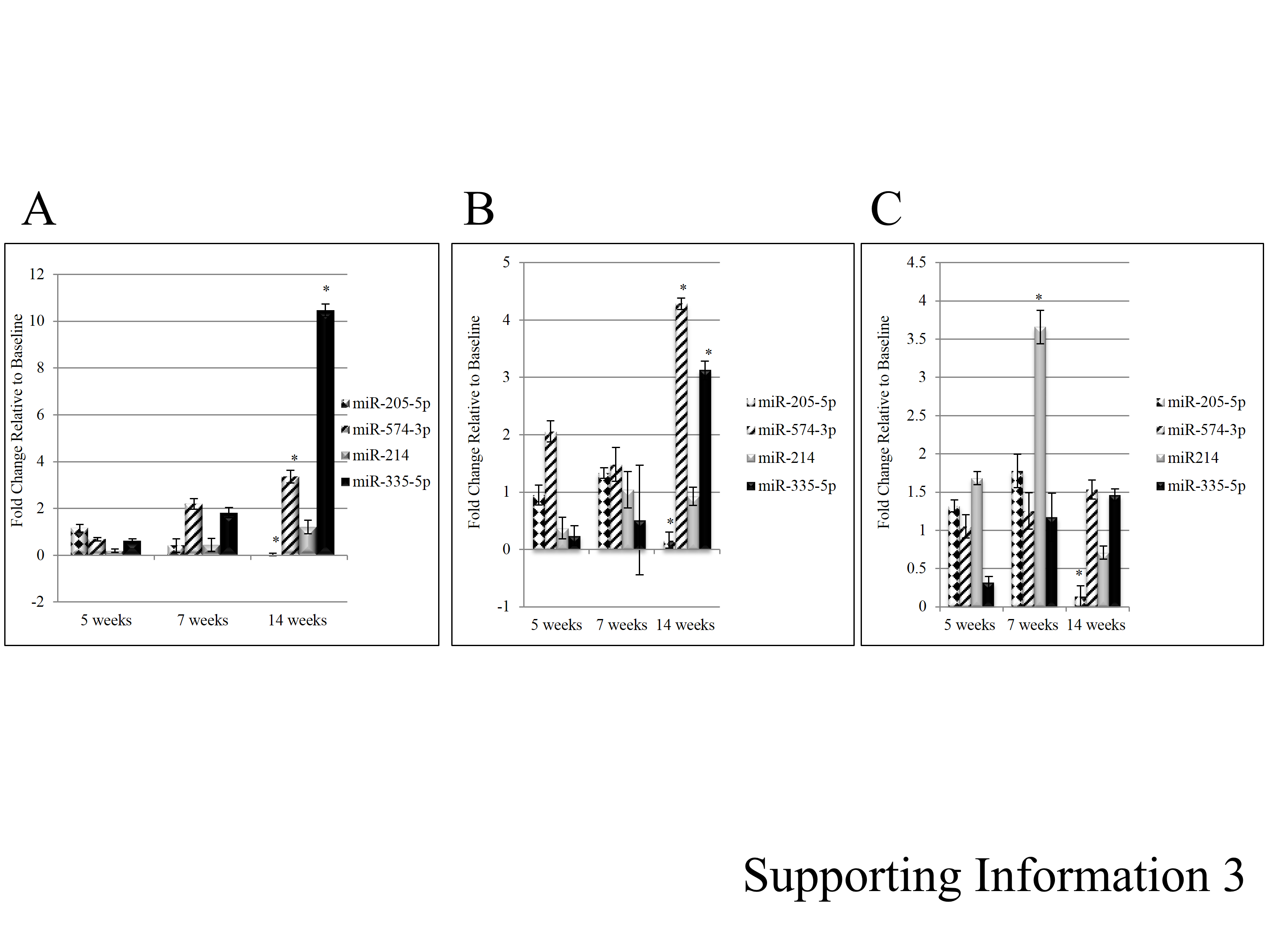

Supplement: Supplementary file 2 [file cam40004-0977-sd2.zip › CAM4_438_SUPP0003-Supp Info 3 FinalHR.TIF]

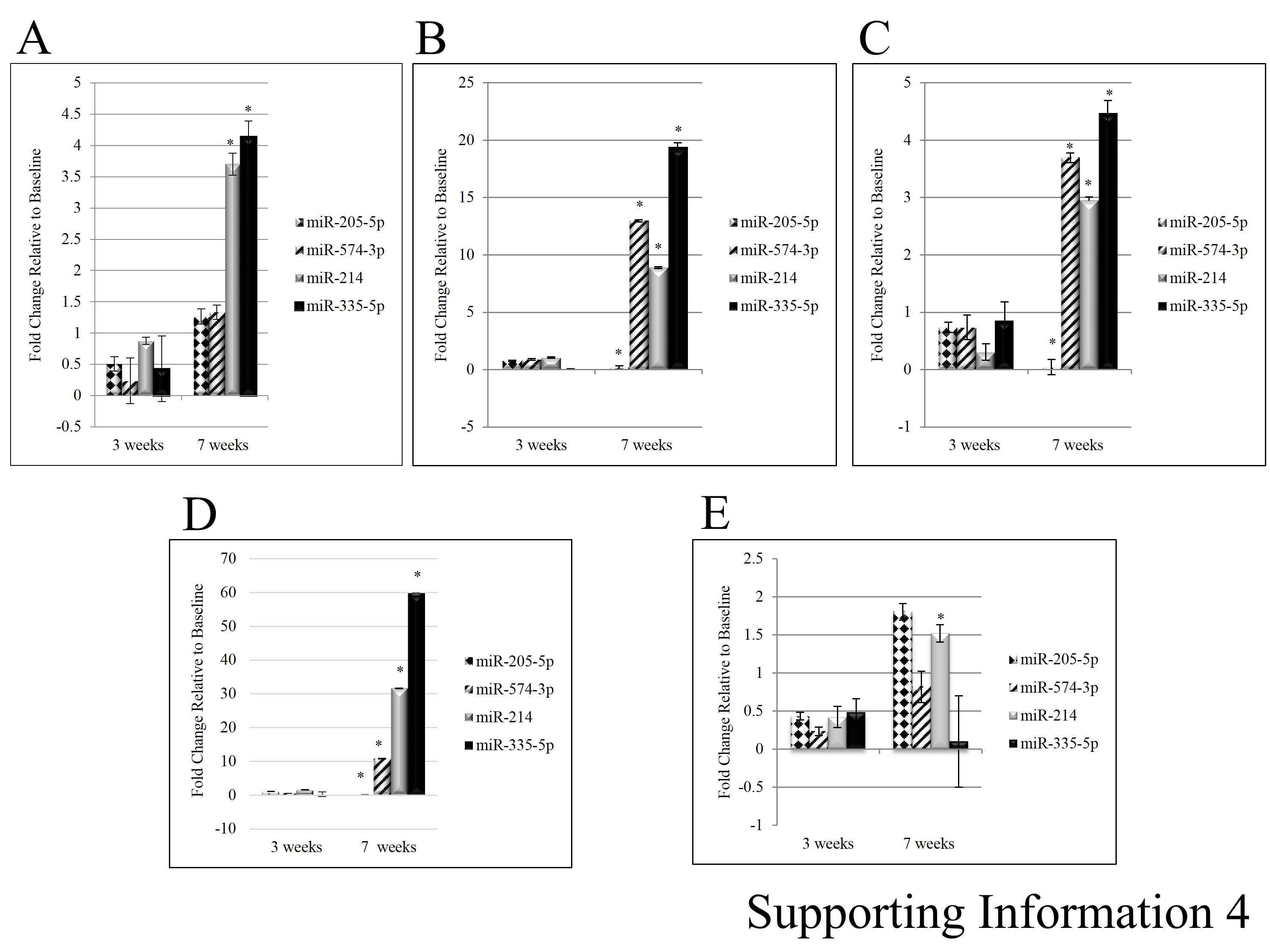

Supplement: Supplementary file 2 [file cam40004-0977-sd2.zip › CAM4_438_SUPP0004-Supp Info 4 FinalHR.TIF]

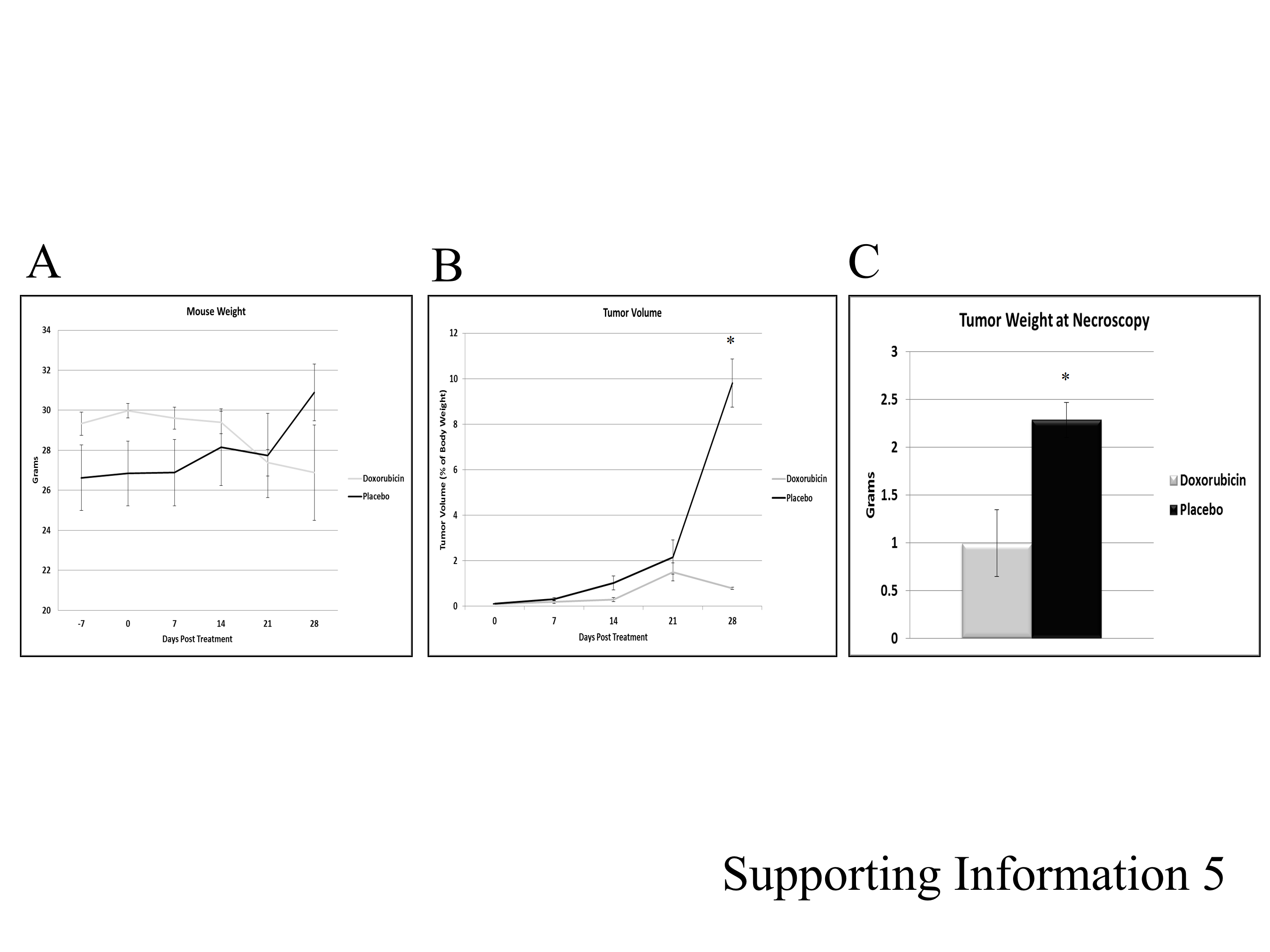

Supplement: Supplementary file 2 [file cam40004-0977-sd2.zip › CAM4_438_SUPP0005-Supp Info 5 FinalHR.TIF]

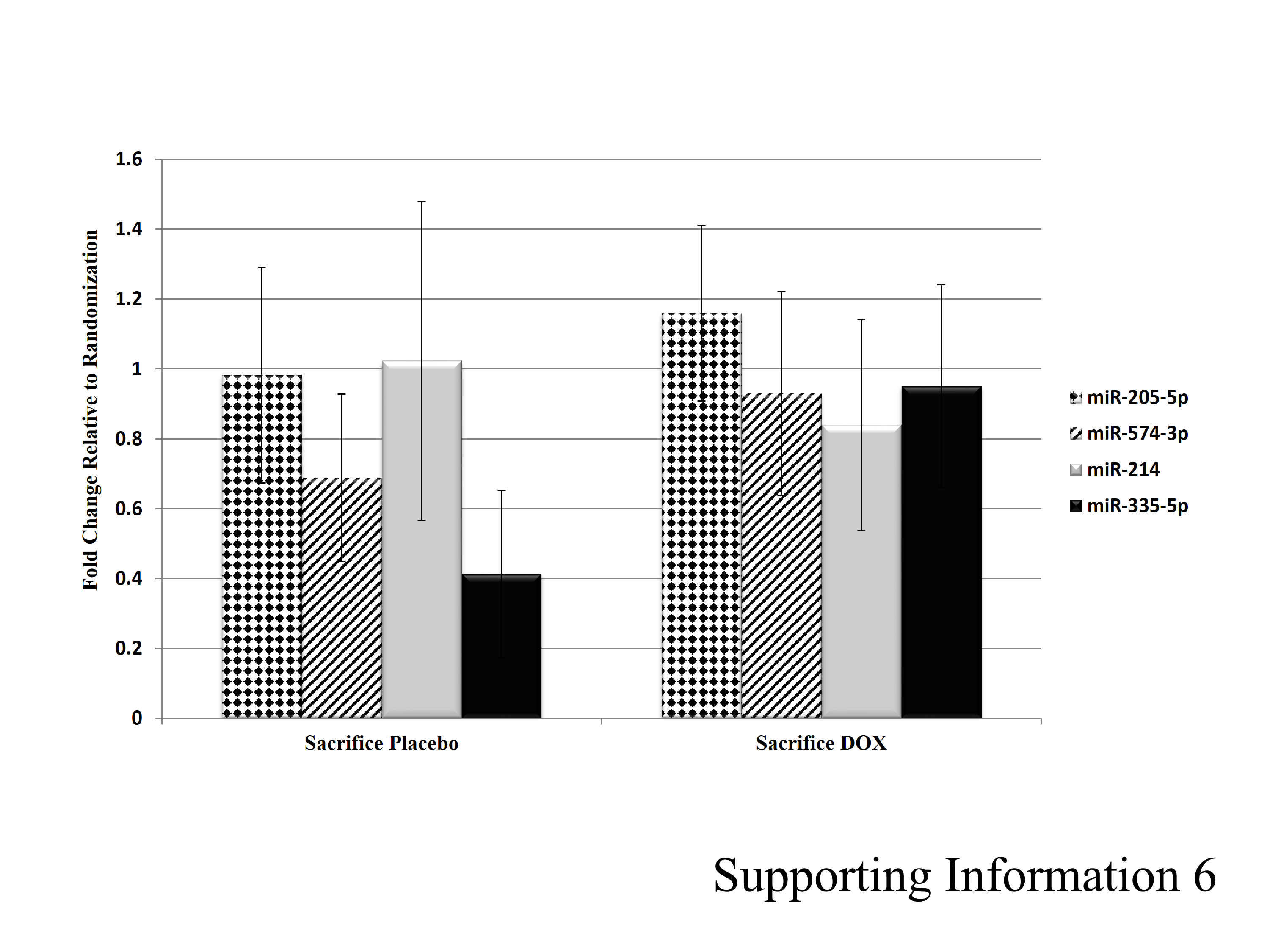

Supplement: Supplementary file 2 [file cam40004-0977-sd2.zip › CAM4_438_SUPP0006-Supp Info 6 FinalHR.TIF]

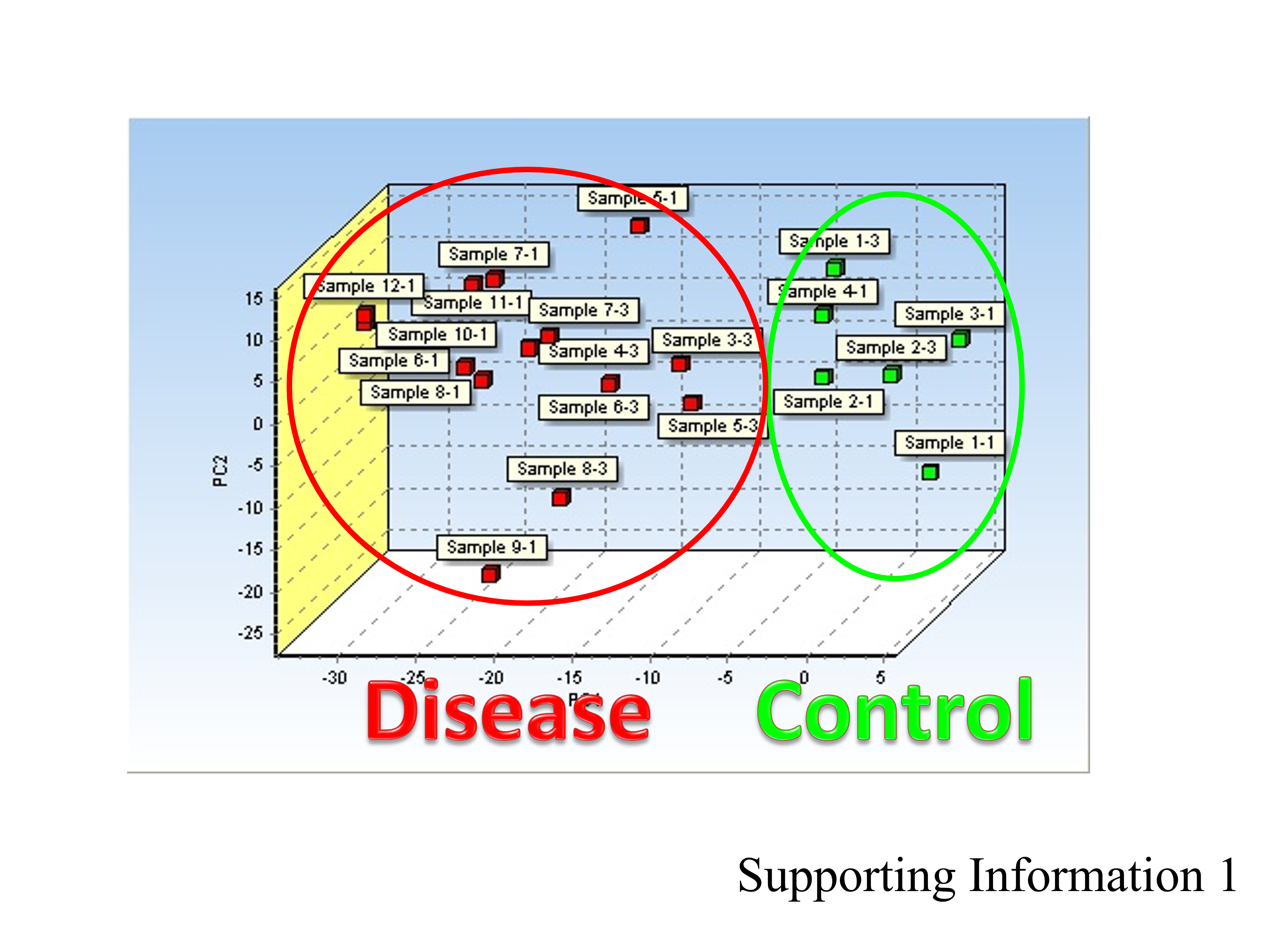

Supplement: Supplementary file 2 [file cam40004-0977-sd2.zip › CAM4_438_SUPP0001-Supp Info 1 FinalHR.tif]
